# Efficacy of a Psychoeducational Program (Cuidando Juntos) to Support Psychological Health of Latino Caregivers

**DOI:** 10.1093/geroni/igaf122.2649

**Published:** 2025-12-31

**Authors:** Julian Montoro-Rodriguez, Charlie Reeve, Dolores Gallagher-Thompson, Roberto Velasquez, Ann Bilbrey, Jennifer Ramsey, Bruno Kajiyama, Maria Quiñones-Cordero

**Affiliations:** The University of North Carolina at Charlotte, Charlotte, North Carolina, United States; The University of North Carolina at Charlotte, Charlotte, North Carolina, United States; Stanford University, Stanford, California, United States; Southern Caregiver Resource Center, Chula Vista, California, United States; Optimal Aging Center, Sunnyvale, California, United States; York Technical College, Rock Hill, South Carolina, United States; Photozig, Inc., Moffett Field, California, United States; University of Rochester School of Nursing, Rochester, New York, United States

## Abstract

Latinos have the highest prevalence of caring for a family member with Alzheimer’s disease and have significantly worse physical and psychological outcomes than other groups. Yet, there are few culturally appropriate evidence-based psychoeducational skill-building programs for Latinos. Our team culturally adapted the online Caregiver TLC skill-building program to support Latino caregivers (Montoro-Rodriguez et al., 2024; 2025). Our goal is to examine the acceptability and efficacy of this adapted program to improve social connectedness and overall health of Latino caregivers: Cuidando Juntos. Using pre-post data from the pilot clinical trial (N = 31), we examined user-reactions to the program to assess some aspects of acceptability, as well as changes on measures of general health, care-giving self-efficacy, depression, perceived stress, and burden to evaluate program efficacy. Data was collected from the community organizations in San Diego, CA and Charlotte, NC. Findings from the analyses indicated significant improvement in scores for all five outcomes from pre- to post-program assessments. Scores on general health and self-efficacy increased, whereas scores on depression, perceived stress, and burden decreased. Effect sizes ranged from small to very large. These results indicate that the pilot program is viewed as an acceptable and useful program by the sample of Latino caregivers attending the program and it has potential for a positive impact on caregivers’ self-reported general and mental health. This pilot findings suggest that Cuidando Juntos may yield mental health benefits to Latino caregivers of older adults with significant memory problems.

